# Overexpression of OAS1 Is Correlated With Poor Prognosis in Pancreatic Cancer

**DOI:** 10.3389/fonc.2022.944194

**Published:** 2022-07-11

**Authors:** Lingling Lu, Huaxiang Wang, Jian Fang, Jiaolong Zheng, Bang Liu, Lei Xia, Dongliang Li

**Affiliations:** ^1^ Fuzong Clinical Medical College of Fujian Medical University, Fuzhou, China; ^2^ Department of Hepatobiliary and Pancreatic Surgery, Taihe Hospital, Affiliated Hospital of Hubei University of Medicine, Shiyan, China; ^3^ Department of Hepatobiliary Medicine, The Third Affiliated People's Hospital of Fujian University of Traditional Chinese Medicine, Fuzhou, China; ^4^ Department of Hepatobiliary Disease, The 900th Hospital of the People’s Liberation Army Joint Logistics Support Force, Fuzhou, China

**Keywords:** pancreatic cancer, prognosis, OAS1, overall survival, immune infiltration

## Abstract

**Background:**

OAS1 expression in pancreatic cancer has been confirmed by many studies. However, the prognostic value and mechanism of OAS1 in pancreatic cancer have not been analyzed.

**Methods:**

The RNA-seq in pancreatic cancer were obtained by UCSC XENA and GEO database. In addition, immunohistochemical validation and analysis were performed using samples from the 900th hospital. The prognosis of OAS1 was evaluated by timeROC package, Cox regression analysis, and Kaplan-Meier survival curves. Then, the main functional and biological signaling pathways enrichment and its relationship with the abundance of immune cells were analyzed by bioinformatics.

**Results:**

OAS1 was highly expressed in pancreatic cancer compared with normal pancreatic tissue. High OAS1 expression was associated with poor overall survival (p<0.05). The OAS1 was significantly correlated to TNM staging (p=0.014). The timeROC analysis showed that the AUC of OAS1 was 0.734 for 3-year OS. In addition, the expression of OAS1 was significantly correlated with the abundance of a variety of immune markers. GSEA showed that enhanced signaling pathways associated with OAS1 include Apoptosis, Notch signaling pathway, and P53 signaling pathway.

**Conclusions:**

OAS1 is a valuable prognostic factor in pancreatic cancer. Moreover, it may be a potential immunotherapeutic target.

## Introduction

Once pancreatic cancer has metastasized, the 5-year survival rate is as low as 2.9% (1). Early surgical intervention is still the radical cure. However, most patients with pancreatic cancer were found late and missed the best time for treatment. This may be related to the lack of early detection and identifiable symptoms and signs (2). With the development of multidisciplinary therapy, patients with pancreatic cancer can achieve better outcomes. However, the overall prognosis is still not satisfactory due to the high tumor-specific mortality rate. and the annual mortality and morbidity rates are similar (3). The prognosis of pancreatic cancer is still worthy of special attention. However, there are many factors affecting the prognosis of patients, including not only the common tumor size, tumor stage (4) and the presence of disseminated tumor cells, but also the systemic inflammatory response (5), which reflects the response of the immune system to the proliferation and survival of tumor cells, which affects the ability of tumor cells to induce angiogenesis and metastasis and spread. In recent years, studies on tumor microenvironment (TME) have confirmed the key role of immune cells in the occurrence and development of cancer. Moreover, gene biomarkers have been widely explored in pancreatic cancer (6).

2’,5’-oligoadenylate synthetase 1 (OAS1), a member of OAS family, has been shown to be a protein family of interferon-induced enzymes (7). The OAS family are involved in many intracellular functions, including induction of apoptosis, enhancement of IFN- α signal response, gene regulation, immune cell receptor regulation, and autophagy (8, 9). Moreover, OAS1 has been shown to be correlated with different subcellular components, such as mitochondria, nuclei, and rough/smooth microsomes (10, 11). It has been found that OAS1 is highly expressed in many tumors (12). And the high expression was related to worse prognosis in breast cancer (13). OAS1 may be associated with gastric cancer resistant to trastuzumab (14). It has been proved that down-regulating the expression of OAS1 can lead to the decrease of cell motility *in vitro* (15). In addition, OAS1 may be regulated by 17β-estradiol (E2) and play a key role in inducing apoptosis in cancer cells (16, 17). It is closely correlated with the occurrence and progress of gastric cancer (14), breast cancer (18), lung adenocarcinoma (19), and bladder cancer (20). However, the relationship between OAS1 and pancreatic cancer has not been clarified. Whether OAS1 can be used as a prognostic indicator of pancreatic cancer is worth exploring.

Here, it is particularly important to find reliable biomarkers related to prognosis in pancreatic cancer. We applied bioinformatics method to analyze the relationship between OAS1 and clinical information and overall survival (OS) of pancreatic adenocarcinoma (PAAD). Moreover, the immune-related database was used to analyze the relationship between OAS1 and tumor microenvironment. The aim was to explore the prognostic value of OAS1 in pancreatic cancer. In addition, Function analysis and GSEA were used to further enrich the signal pathways related to OAS1.

## Materials and Methods

### Ethical Statement

All patients were included in the public database only after obtaining informed consent. Since all the research data are from open online databases, all informed consent can be guaranteed.

### Differential Expression of OAS1

UCSC XENA (https://xenabrowser.net/datapages/) is a data analysis platform for cancer genomics, which includes data from TCGA, ICGC, and other projects. It can provide online analysis and data visualization. TCGA and GTEx RNAseq data in TPM format are processed through toil (21). This study extracted data of pancreatic adenocarcinoma (PAAD) from TCGA and corresponding normal tissue from GTEx. The R statistical language (version 3.6.3) and the ggplot2 package were used to analyze mRNA differential expression and for plotting. The GSE15471 dataset in the GEO (22) database was also used to evaluate the difference in OAS1 expression between pancreatic cancer and normal tissue. The Human Protein Atlas (https://www.proteinatlas.org/) (23) provides proteomic and transcriptome information for a variety of human samples, including monocytic, blood, tissue, and pathological maps. The database was applied to obtain the protein immunohistochemistry (IHC) of OAS1 in normal tissues and pancreatic cancer tissues.

### Immunohistochemistry and Immunofluorescence

45 cases of pancreatic cancer tissue specimens were embedded in paraffin and cut into 4 μm sections. Next, the slides were then dewaxed with xylene at room temperature and then hydrated with degraded ethanol. Steam 20 minutes in sodium citrate buffer (PH 6.0) for antigen retrieval. H2O2 was used to inhibit endogenous peroxidase. The polyclonal rabbit anti-OAS1 antibody (1:100, LS-B6622, LifeSpan Biosciences) was added to the slices, incubated at room temperature for 1 hour, and washed in PBS for 3 times. Then, secondary antibody (PV-9001; goat anti-rabbit IgG polymer; Beijing Zhongshan Jinqiao Biotechnology Co., LTD) was added at room temperature and incubate for 30 minutes. Finally, diaminobenzidine (DAB) was used for 1 minute at room temperature, and the slides were stained with hematoxylin. We used a semi-quantitative scoring system to assess OAS1 protein expression. Score of 0 indicates no positive cells, while a score of 1 indicates less than 10% positive cells, 2 indicates 11%-25% positive cells, 3 indicates 26-50% positive cells, 4 indicates 51-75% positive cells, and 5 indicates greater than 75% positive cells. The scores of 0, 1 and 2 indicate the low expression of OAS1, while the higher the score, the higher the expression of OAS1. The score of IHC staining was performed by two experienced pathologists without knowing the patient’s clinical information. All samples were taken from the 900th hospital of the People’s Liberation Army Joint Logistics Support Force (Fujian, China). This study was approved by the Ethics Committee of the 900th hospital of the People’s Liberation Army Joint Logistics Support Force and carried out in accordance with the Helsinki Declaration.

Similarly, paraffin blocks of pancreatic cancer were sectioned for dewaxing, rehydration, and antigen retrieval. They were then blocked with PBST containing 5% FBS for 30 min. Next, primary antibody (1:100, LS-B6622, LifeSpan Biosciences) was dropped and incubated at room temperature for 2 hours. Then the slides were washed with PBST and incubated with secondary antibody (1:1000, 8889s, CST) for 2 hours at room temperature. Finally, 4, 6-Diamidino-2-phenylindole (DAPI) (C1005, Beyotime Biotechanology, China) was stained for 4 minutes and observed by confocal laser scanning microscope.

### Prognostic Value of OAS1

Extract the RNAseq data and clinical data from the TCGA-PAAD project, and eliminate the data with missing clinical information. According to the median value of OAS1, they were divided into two groups: high expression group and low expression group. We used Kaplan-Meier survival curves to compare survival differences, and to study the relationship between OAS1 expression level and clinical outcome. Then using the survminer and survival package for statistical analysis of survival data. In addition, the independent prognostic factors were determined by Cox regression analysis. Using timeROC package to draw receiver operating characteristic (ROC) curve to evaluate the accuracy of OAS1 in survival prediction. OS is defined as the time between first diagnosis and death or the last observation point. Disease specific survival (DSS) was defined as the period from the date of diagnosis of pancreatic cancer to the recorded date of death due to pancreatic cancer. Progression free interval (PFI) is defined as the time between the start of follow-up and the first appearance of progression (24).

### Immune Infiltration Analysis and Gene Alterations

TIMER (https://cistrome.shinyapps.io/timer/) was applied to analyze the relationship between gene expression and immune infiltrate or abundance online (25). In this work, TIMER data was used to identify the relationship between the expression of OAS1 and immune cells infiltration in pancreatic cancer. Furthermore, TISIDB (http://cis.hku.hk/TISIDB/) (26) was conducted to analyze the relationship between abundance of tumor-infiltrating lymphocytes (TILs) and expression of OAS1. Then for genetic changes of OAS1 gene, the data of cBioPortal (http://www.cbioportal.org/) (27) database were inquired, and the relationship between genetic alterations and clinical outcome, including OS and DFS, was obtained.

### Functional Analysis and PPI Network Construction

The top 100 genes with the strongest correlation with OAS1 were found by using GEIPIA (http://gepia.cancer-pku.cn/index.html) (28) database and cBioportal database respectively, and the intersection was taken. Then, GO and KEGG (29) enrichment analysis of OAS1 related genes was carried out by using functional annotation tool in DAVID (https://david.ncifcrf.gov/) (30) database. In the process of GO and KEGG enrichment analysis, false discovery rate (FDR) < 0.5 or P<0.05 was used as the screening condition. In addition, the protein-protein interaction (PPI) network of OAS1-related genes was constructed in STRING (https://string-db.org/) (31) database and visualized in Cytoscape software (Version 3.7.1).

### Gene Set Enrichment Analysis(GSEA)

GSEA is executed using GSEA software (version 4.0.3). Biological pathways associated with OAS1 were detected by gene enrichment analysis (GSEA). All the genetic data analyzed were obtained from TCGA database.

## Results

### Elevated Expression of OAS1 in PAAD

**Figure 1:**
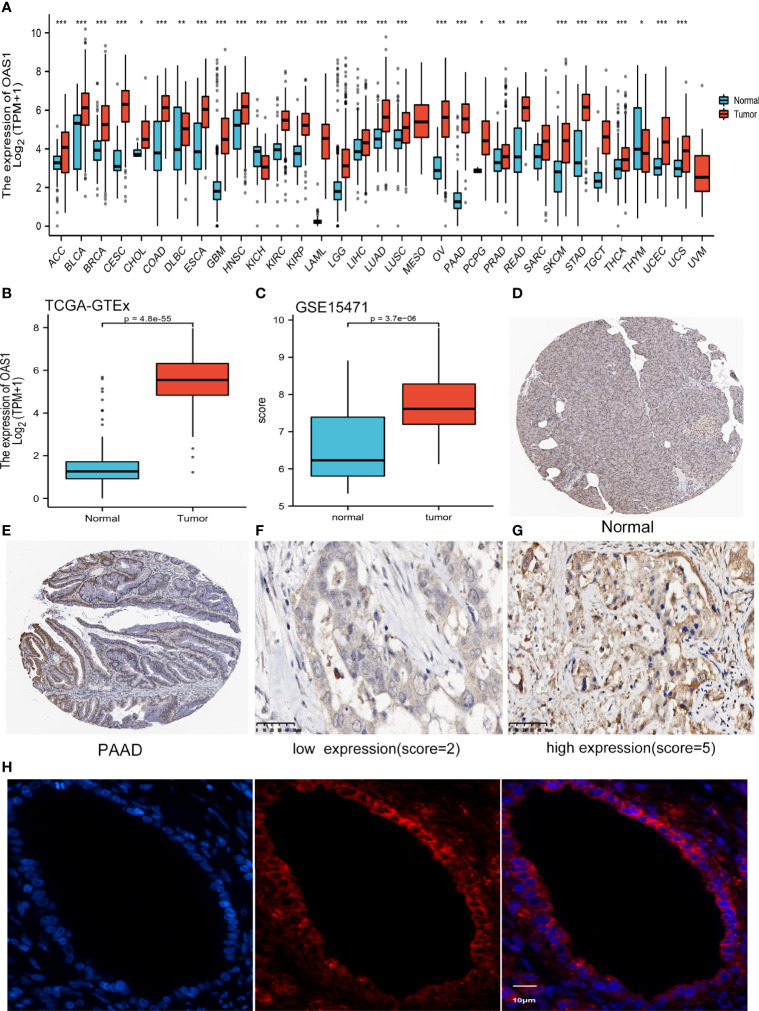
The expression of OAS1 **(A)** OAS1 expression in different types of cancers (*P < 0.05, **P <0.01, ***P < 0.001). **(B)** OAS1 expression in pancreatic cancer and normal pancreatic tissue base on TCGA-PAAD. **(C)** OAS1 expression in pancreatic cancer and normal tissue base on GSE15471 of GEO. **(D, E)** The protein levels of OAS1 between normal pancreatic tissue and pancreatic cancer tissue based on HPA. **(F, G)** Representative Images of low **(F)** /High **(G)** protein expression of OAS1 in 45 patients with PAAD (original magnification:X400). **(H)** Localization of OAS1 (red) was observed by immunofluorescence confocal microscopy. The nuclei were stained (blue) with DAPI. Representative pictures are displayed.

We preliminarily evaluated the transcription levels of OAS1 in different human tumors by analyzing TCGA and GTEx RNA-seq data using the UCSC XENA database. OAS1 was highly expressed in 29 kinds of tumors including PAAD. It is interesting to note that OAS1 was only found to be significantly lower expressed in the tissues of kidney chromophobe (KICH) and Thymoma (THYM) than in the respective control tissues (**Figure 1A**). These results suggest that OAS1 is abnormally high expressed in most tumors. Then we compared OAS1 mRNA expression in 179 pancreatic cancer tissues and 171 normal tissues. The expression of OAS1 mRNA in PAAD tissue was significantly higher than that in normal pancreatic tissue (p<0.001, **Figure 1B**). In addition, we further analyzed the expression level of OAS1 in pancreatic cancer tissue using GEO data (GSE15471), and found that OAS1 mRNA in tumors was also significantly higher than that in normal tissue (p<0.001, **Figure 1C**). Overall, these results suggest that elevated expression of OAS1 in PAAD than in normal tissues.

We compared the protein expression of OAS1 between normal pancreatic tissue and pancreatic cancer tissue by HPA. As shown in **Figures 1D, E**, immunohistochemical staining also indicated OAS1 was upregulated in pancreatic cancer tissue.

### OAS1 and the Clinicopathological Features in the Patients From the 900th Hospital

**Figure 2:**
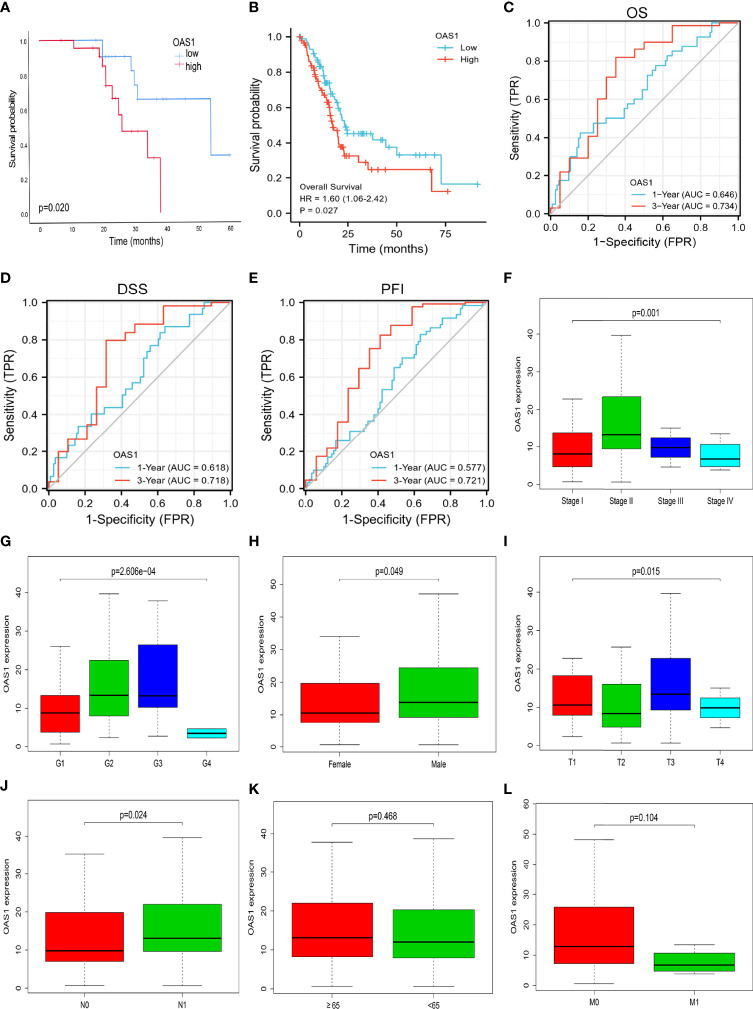
**(A, B)** Kaplan-Meier survival curves for OAS1, and high expression of OAS1 has short overall survival in PAAD. The time-dependent ROC to assess the accuracy of OAS1 in predicting OS **(C)**, DSS **(D)**, and PFI **(E)** at 1 and 3 years. The relationships between OAS1 mRNA expression and clinical pathological characteristics. OAS1 mRNA expression was differentially expressed in different TNM stages **(F)**, grades **(G)**, gender **(H)**, T stages **(I)**, and N stages **(J)**. There was no difference in ages or M stages **(K, L)**.

A total of 45 cases of pancreatic cancer samples from the 900th hospital of the People’s Liberation Army Joint Logistics Support Force were collected. There were 14 females and 31 males. Mean age was 59.22 years (range 41-82). According to semi-quantitative scoring system, 45 cases of pancreatic cancer were divided into high expression group (n=23) and low expression group (n=22). The median overall survival was 21.0 months and 23.0 months for patients with high and low OAS1 expression, respectively. And the mean value of overall survival was 21.12 months and 25.18 months for patients with high and low OAS1 expression, respectively. As shown in **Figures 1F, G**, OAS1 expression was divided into high and low groups, and it was mainly expressed in the cytoplasm of pancreatic cancer cells. The immunofluorescence results showed that OAS1 was mainly observed in cytoplasm (**Figure 1H**). Furthermore, Kaplan-Meier survival curves showed that the high expression of OAS1 was related to the poor OS (p=0.020) in patients with pancreatic cancer (**Figure 2A**).

### Survival Analysis

RNAseq data and clinical data from the TCGA-PAAD project were obtained for analysis. Samples are grouped according to the median value of OAS1 and then to study the correlation between the expression of OAS1 and the prognosis of patients with pancreatic cancer. Then we summarize the basic clinical features of the patient in **Table 1**. Of the 178 patients, 176 underwent surgery, including Whipple, total pancreatectomy, distal pancreatectomy, and other method. And postoperative residual tumors status can be obtained in 164 cases. Only one patient received neoadjuvant therapy. 118 out of the 178 patients received chemotherapy. The patients were treated with drugs, including “Gemcitabine”, “Xeloda”, “5-FU”, “Tarceva”, “Capecitabine”, “Irinotecan”. “Oxaliplatin”, “Leucovorin”, “Abraxane”, “Cisplatin”, “Docetaxel” “cyclophosphamide”, and “gemzar”. In addition, 45 of the 178 patients received radiation therapy. The median survival time in individuals with low OAS1 expression was 23.4 years in comparison with the median survival time of 17.2 years in high OAS1 expression. Moreover, Kaplan-Meier survival curves showed that the prognostic value of OAS1 in pancreatic cancer (**Figure 2B**). The results indicated that the high expression of OAS1 was significantly correlated with the shorter OS of PAAD (HR 1.60, 95%CI,1.06-2.242; p=0.027). But we observed no significant relationship between OAS1 expression and disease-free survival (p=0.17). The timeROC analysis showed that the AUC of OAS1 was 0.646 and 0.734 for 1- and 3-year OS, respectively (**Figure 2C**). The AUC for 1- and 3-year DSS were 0.618 and 0.717, respectively (**Figure 2D**), and the AUC for 1- and 3-year PFI were 0.577and 0.721, respectively (**Figure 2E**). These results showed the moderate predict performance of OAS1 and which is limited to 3-year OS, DSS, and PFI.

**Table 1:** Baseline characteristics for patients with pancreatic cancer from TCGA.

Characteristic	Levels	Overall
n		178
T stage, n (%)	T1	7 (4%)
	T2	24 (13.6%)
	T3	142 (80.7%)
	T4	3 (1.7%)
N stage, n (%)	N0	50 (28.9%)
	N1	123 (71.1%)
	N2	0
M stage, n (%)	M0	79 (94%)
	M1	5 (6%)
Pathologic stage, n (%)	Stage I	21 (12%)
	Stage II	146 (83.4%)
	Stage III	3 (1.7%)
	Stage IV	5 (2.9%)
Gender, n (%)	Female	80 (44.9%)
	Male	98 (55.1%)
Race, n (%)	Asian	11 (6.3%)
	Black or African American	6 (3.4%)
	White	157 (90.2%)
Age, n (%)	<=65	93 (52.2%)
	>65	85 (47.8%)
Residual tumor, n (%)	R0	107 (65.2%)
	R1	52 (31.7%)
	R2	5 (3.1%)
Chemotherapy	No/unkown	60 (33.7%)
	Yes	118 (66.3%)
Radiation therapy, n (%)	No	118 (72.4%)
	Yes	45 (27.6%)
Histologic grade, n (%)	G1	31 (17.6%)
	G2	95 (54%)
	G3	48 (27.3%)
	G4	2 (1.1%)
Anatomic neoplasm subdivision, n (%)	Head of Pancreas	139 (78.1%)
	Other	39 (21.9%)
Smoker, n (%)	No	65 (45.1%)
	Yes	79 (54.9%)
Alcohol history, n (%)	No	65 (39.2%)
	Yes	101 (60.8%)
History of diabetes, n (%)	No	108 (74%)
	Yes	38 (26%)
History of chronic pancreatitis, n (%)	No	128 (90.8%)
	Yes	13 (9.2%)
OS event, n (%)	Alive	86 (48.3%)
	Dead	92 (51.7%)
DSS event, n (%)	Alive	100 (58.1%)
	Dead	72 (41.9%)
PFI event, n (%)	Alive	74 (41.6%)
	Dead	104 (58.4%)

### Correlation Between OAS1 Expression and Clinicopathological Parameters

We obtained clinical data from the TCGA-PAAD project and studied the relationship between OAS1 and clinicopathological features, which is helpful to reveal the role of OAS1 in the progression of PAAD. As shown in **Figures 2F–L**, OAS1 mRNA expression was differentially expressed in different grades (p<0.001), gender (p=0.049), T stages (p=0.015), N stages (p=0.024), and TNM stages (p=0.001). There was no difference in age or M stage. Moreover, the Cox regression analysis was applied to analyze the prognostic factors. As shown in **Table 2**, the univariate analysis indicated that high OAS1 expression was correlated with the poorer OS (HR 1.545, 95%CI: 1.021-2.338, p=0.040). Other clinical parameters including T stage (HR 2.023, 95%CI: 1.072-3.816, p=0.030), and N stage (HR 2.154, 95%CI: 1.282-3.618, p=0.004) were also associated with the poorer overall survival. Multivariate analysis showed that only N staging (HR 1.870, 95%CI:1.079-3.240, p=0.026) was independently associated with OS.

**Table 2:** Univariate/multivariate Cox regression analysis.

		Univariate analysis		Multivariate analysis	
Characteristics	Total (N)	HR (95% CI)	P value	HR (95% CI)	P value
Gender (Male vs. Female)	178	0.809 (0.537-1.219)	0.311		
Age (>65 vs. <=65)	178	1.290 (0.854-1.948)	0.227		
Smoker (No vs. Yes)	144	0.921 (0.582-1.456)	0.724		
History of diabetes (No vs. Yes)	146	1.078 (0.619-1.878)	0.790		
T stage (T3&T4 vs. T1&T2)	176	2.023 (1.072-3.816)	**0.030**	1.261 (0.651-2.442)	0.492
N stage (N1 vs. N0)	173	2.154 (1.282-3.618)	**0.004**	1.870 (1.079-3.240)	0.026
M stage (M1 vs. M0)	84	0.756 (0.181-3.157)	0.701		
OAS1 (High vs. Low)	178	1.545 (1.021-2.338)	**0.040**	1.174 (0.768-1.795)	0.458

Bold values means that the P value < 0.05 were considered statistically significant.

### Gene Alterations in OAS1 and Survival

**Figure 3:**
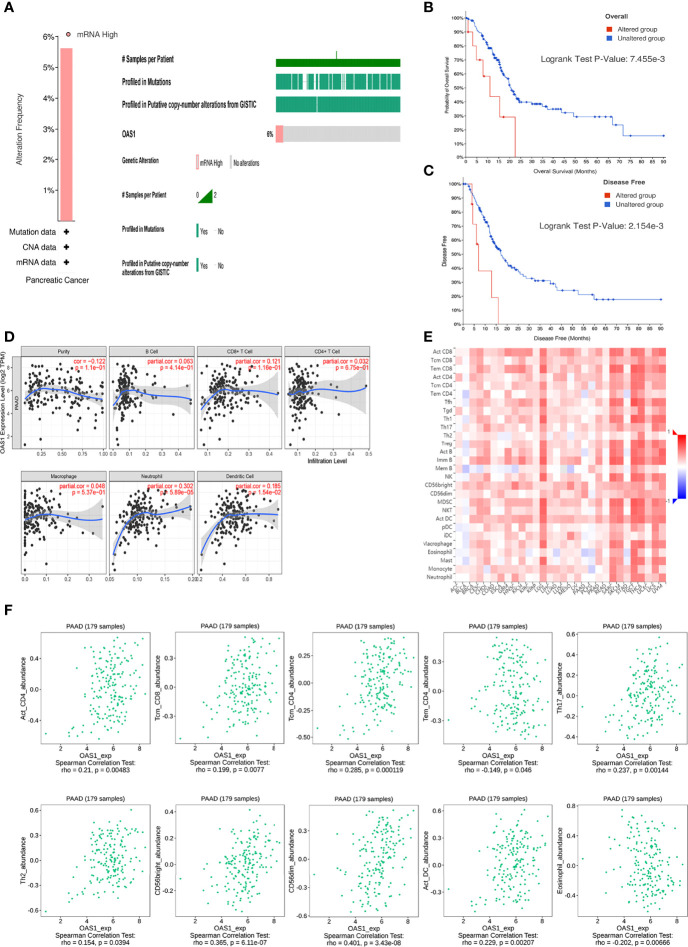
Genetic alterations and immune infiltration. **(A)** Genetic alterations of OAS1 in patients with pancreatic cancer. The patient of altered group received a shorter OS **(B)** and DFS **(C)** than that of unaltered group. **(D)** OAS1 expression had correlations with neutrophil and dendritic cell. **(E)** correlations of OAS1 expression with TILs across human cancers. **(F)** The expression of OAS1 was associated with activated CD4 T cell, central memory CD8 T cell, central memory CD4 T cell, effector memory CD4 T cell, Type 17 T helper cell, Type 2 T helper cell, CD56bright natural killer cell, CD56dim natural killer cell, activated dendritic cell, and eosinophil.

We analyzed the gene alterations of OAS1 and their relationship with OS and DFS. As shown in **Figure 3A**, 10 of the 178 sequenced patients (5.62%) had gene alterations. In addition, the patient of altered group received a shorter OS (p<0.01; **Figure 3B**) and DFS (p<0.01; **Figure 3C**) than that of unaltered group. These results suggest that gene alteration of OAS1 may also significantly affect the prognosis of patients with pancreatic cancer.

### OAS1 and Immune Cell Infiltration

TIMER database was used to investigate the correlation between the expression of OAS1 and immune cell infiltration in patients with PAAD. OAS1 expression had correlations with neutrophil (cor=0.302, p<0.001) and dendritic cell (cor=0.185, p<0.05) (**Figure 3D**). It highlights the key role of OAS1 in tumor immune infiltrating cells, neutrophils, and dendritic cells.

We also evaluated the relations between OAS1 expression and tumor-infiltrating lymphocytes (TILs) in the TISIDB database (**Figures 3E, F**). The expression of OAS1 was associated with activated CD4 T cell (rho=0.21, p<0.01), central memory CD8 T cell (rho=0.199, p<0.01), central memory CD4 T cell (rho=0.285, p<0.001), effector memory CD4 T cell (rho=-0.149, p<0.05), Type 17 T helper cell (rho=0.237, p<0.01), Type 2 T helper cell (rho=0.154, p<0.05), CD56bright natural killer cell (rho=0.365, p<0.001), CD56dim natural killer cell (rho=0.401, p<0.001), activated dendritic cell (rho=0.229, p<0.01), and eosinophil (rho=-0.202, p<0.01). Those results revealed that OAS1 may play a specific role in the immune infiltration of pancreatic cancer.

### Functional Analysis and PPI Network Construction

**Figure 4:**
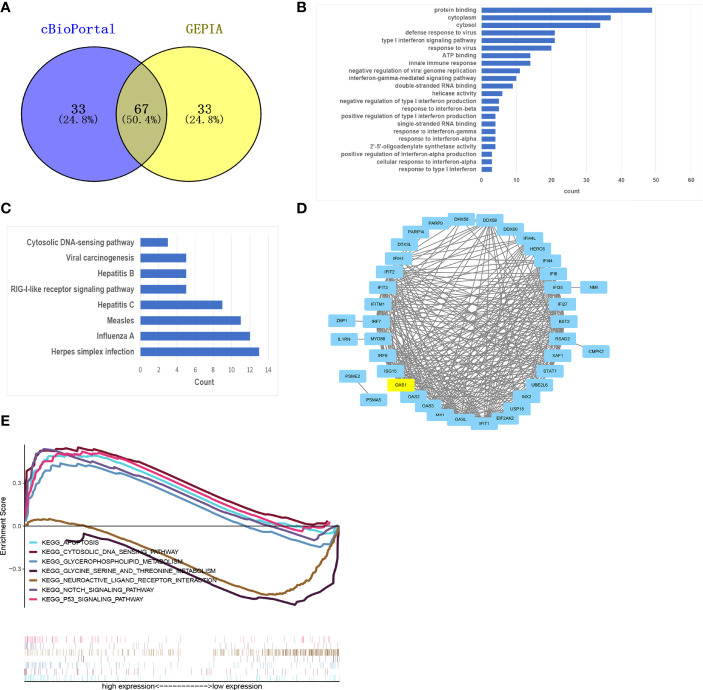
Functional analysis and GSEA. **(A)** Venn plot showed overlapping genes in GEPIA and cBioPortal databases. **(B, C)** The 67 OAS1-related genes were analyzed by GO **(B)** and KEGG **(C)** enrichment analysis. **(D)** A PPI network of 67 OAS1-related genes was constructed and visualized. **(E)** GSEA identifies OAS1-related signaling pathways in pancreatic cancer.

Using cBioPortal and GEPIA database to find the top 100 genes with the strongest correlation with OAS1, and taking the intersection, 67 related genes can be obtained (**Figure 4A**). Then the 67 genes were analyzed by GO enrichment analysis including BP, CC, and MF (**Supplementary table 1**). GO-MF analysis showed that the related genes were most enriched by protein binding, ATP binding, and double-stranded RNA binding. Then visualize all the GO analysis results, as shown in **Figure 4B**. We also analyzed the related genes by KEGG enrichment analysis. In KEGG analysis, Cytosolic DNA-sensing pathway, RIG-I-like receptor signaling pathway, and Herpes simplex infection were enriched (**Figure 4C**). RIG-I-like receptor signaling pathway has been confirmed to be involved in the occurrence and development of tumors. The enrichment of the Herpes simplex infection suggests that the tumor may simulate immune mechanisms following viral infection. Finally, the interaction between these proteins is shown in **Figure 4D**.

### GSEA Identifies OAS1-Related Signaling Pathways in Pancreatic Cancer

To explore the potential molecular function of OAS1 in pancreatic cancer, we conducted GSEA between low and high expression samples to predict OAS1-related signaling pathways. A total of 131 out of 178 gene sets are upregulated in the group of high OAS1 expression, and 47 out of 178 gene sets are upregulated in the group of low OAS1 expression. But only 20 signaling pathways were significantly enriched at NOM P < 0.05 (**Table 3**). Inhibitory signaling pathways associated with OAS1 include “Neuroactive ligand receptor interaction” and “Glycine serine and threonine metabolism”. Enhanced signaling pathways associated with OAS1 include “Apoptosis”, “Cytosolic and sensing pathway”, “Glycerophospholipid metabolism”, “Notch signaling pathway”, and “P53 signaling pathway”. The results of the GSEA are summarized in **Figure 4E**.

**Table 3:** Enrichment plots by GSEA.

Name	SIZE	ES	NES	NOM p-val	FDR q-val
KEGG_PROTEASOME	46	0.753654	1.784794	0.001930502	0.2470178
KEGG_BASE_EXCISION_REPAIR	35	0.74339	1.922512	0.001945525	0.06864582
KEGG_PATHOGENIC_ESCHERICHIA_COLI_INFECTION	56	0.61267	1.743762	0.002	0.2529717
KEGG_RIG_I_LIKE_RECEPTOR_SIGNALING_PATHWAY	70	0.538909	1.685073	0.008316008	0.22338139
KEGG_NOTCH_SIGNALING_PATHWAY	47	0.538491	1.704543	0.010288066	0.2797902
KEGG_CYTOSOLIC_DNA_SENSING_PATHWAY	54	0.550796	1.691296	0.012320329	0.25508887
KEGG_GLYCOSPHINGOLIPID_BIOSYNTHESIS_LACTO_AND_NEOLACTO_SERIES	26	0.518956	1.593648	0.014314928	0.3099594
KEGG_DRUG_METABOLISM_OTHER_ENZYMES	51	0.527769	1.581207	0.017045455	0.31012228
KEGG_P53_SIGNALING_PATHWAY	68	0.521419	1.658908	0.022044089	0.24438126
KEGG_PORPHYRIN_AND_CHLOROPHYLL_METABOLISM	40	0.526058	1.552143	0.023166023	0.32484913
KEGG_GLYCEROPHOSPHOLIPID_METABOLISM	77	0.438724	1.551786	0.023952097	0.3020833
KEGG_APOPTOSIS	87	0.502444	1.526006	0.026262626	0.2956351
KEGG_FC_GAMMA_R_MEDIATED_PHAGOCYTOSIS	96	0.505976	1.598625	0.034	0.37282383
KEGG_SPLICEOSOME	127	0.564458	1.598448	0.038240917	0.33185923
KEGG_ENDOCYTOSIS	181	0.426483	1.490546	0.03846154	0.30282775
KEGG_ARGININE_AND_PROLINE_METABOLISM	54	0.440407	1.475367	0.03960396	0.29238334
KEGG_PYRIMIDINE_METABOLISM	98	0.499027	1.565153	0.040462427	0.31966165
KEGG_RETINOL_METABOLISM	64	0.49851	1.493813	0.04423077	0.31135574
KEGG_NEUROACTIVE_LIGAND_RECEPTOR_INTERACTION	271	-0.48356	-1.71054	0.001992032	0.3879316
KEGG_GLYCINE_SERINE_AND_THREONINE_METABOLISM	31	-0.57781	-1.6636	0.016632017	0.30423322

## Discussion

With the application of high-throughput sequencing, more and more studies have been conducted on the prognostic value of differential genes in pancreatic cancer in recent years. Song et al. found that the pyrolysis-related gene CASP4 can lead to the progression of pancreatic cancer by promoting fatty acid synthesis and accumulation (32). Other markers, such as AQP5, CDC25C, and TMEM170B, have been proved to be associated with the prognosis of pancreatic cancer (33–35). A specific single gene, or a group of genes, can predict the prognosis of the tumor in a corresponding way. The prediction model based on MCOLN1, PKD1, TRPC3, and TPRC7 can also predict the prognosis of pancreatic cancer (36). Zhang et al. found that most of the 21 immune-related prognostic genes of pancreatic cancer, including OAS1, were up-regulated in pancreatic cancer. OASL, as the most significant positive correlation with the expression of OAS1, was conformed that the high expression of OASL was also associated with poor OS in pancreatic cancer. This further speculates that OAS1 is related to the prognosis of pancreatic cancer (37).

In this study, OAS1 mRNA was significantly upregulated in patients with pancreatic cancer. Kaplan-Meier survival curve and univariate analysis showed that elevated OAS1 was associated with shorter OS. In addition, OAS1 has moderate predictive value in OS, DSS, and PFI at 3 years. The AUC of OAS1 was 0.734, 0.718, and 0.721 for 3-year OS, DSS, and PFI, respectively. Although the AUC was lower than that reported of PSMD6 (AUC=0.887) (38), it is higher than that of EIF4G1 (AUC=0.696) for 3-year OS (39). Furthermore, it has been reported that the gene prediction model performed well in predicting OS (AUC=0.680) and DSS (AUC=0.739) (36). Another model based on five prognostic molecules had AUC values of 0.72 and 0.67 at 1-and 2-years, respectively (40). Previous studies have reported the predictive value of a risk score constructed by combining OAS1 with 20 other prognostic genes in pancreatic cancer, and ROC evaluation showed good predictive value (AUC=0.833) (37).

Hence, OAS1 could be a biomarker for poor prognosis of patients with pancreatic cancer. In addition, it was found that OAS1 may play a key role in the immune infiltration of pancreatic cancer. Therefore, this study suggests that OAS1 may serve as a new prognostic biomarker for pancreatic cancer.

Recent studies have shown that elevated OAS1 is associated with short survival in many cancers, and it is an important biomarker of poor prognosis (13). Our study also found high expression of OAS1 in pancreatic cancer, consistent with those reports of abnormal expression of OAS1 in various cancers (13, 41). In breast cancer, elevated OAS1 is associated with poor prognosis. Qu et al. found that OAS1 is one of the high-risk genes for bladder cancer (42). Mandal et al. confirmed that the SNP of OAS1 rs2660 was associated with prostate cancer (43). Hence, OAS1 may play a carcinogenic or tumor suppressor role, affecting the development of cancers. However, the prognostic value of OAS1 in pancreatic cancer has not been studied. We speculate that OAS1 may be involved in the development of pancreatic cancer. Kaplan-Meier survival curves and univariate analysis were applied, the survival time of pancreatic cancer patients with high OAS1 expression was shorter than that of patients with low OAS1 expression. Therefore, we could speculate that OAS1 can be an important biomarker for poor prognosis of pancreatic cancer.

An important feature of PAAD microenvironment is its dense immunosuppressive stroma, which limits the infiltration of immune cells and therapeutic drug (44). Pancreatic ductal adenocarcinoma tumor cells usually account for only a small part of the tumor. Also, there are heterogeneous cell groups in TME, including immune cells, cancer-related fibroblasts (45), endothelial cells and neurons. As shown in **Figure 1**, OAS1 was also expressed in the stroma, especially in tumors with high expression of OAS1. But it is not clear what type of cell in the stroma is expressed. The dense stroma breaks down the tumor vascular system, limiting the delivery of nutrients and drugs (46, 47). This may also promote the insensitivity of pancreatic cancer to chemotherapy. Immune infiltration analysis revealed that OAS1 expression was associated with neutrophils and dendritic cells. This is consistent with previous findings that neutrophils are the predominant immune infiltrating cell type associated with the OAS family (13). Neutrophils are involved in tumor metastasis through PI3K-Akt, cytokines (48), and circulating tumor cells-neutrophils cluster pattern (49). Furthermore, in our study, OAS1 was found to be associated with the expression of a variety of tumor-infiltrating immune cells, which is consistent with a recent study (50). These results indicated a possible correlation between OAS1 and immune infiltration in pancreatic cancer. Nevertheless, further studies are needed to verify.

Extensive DNA damage can lead to excessive synthesis of PAR, leading to energy depletion and/or activation of PAR-dependent programmed cell death pathways leading to cell death (12). And the high expression of OAS1 in cancer cells prevents cell death by inhibiting PAR synthesis and promotes their ability to survive DNA damage. Our study found that many OAS1-related pathways are involved in the development and progression of cancer. Those include the notch signaling pathway, and the P53 signaling pathway. However, OAS1 is not directly involved in these two pathways, and the specific mechanisms by which OAS1 affects these pathways remain unclear. The specific mechanism of OAS1 in the pathway needs to be verified in experiments. Although more and more studies have been conducted on OAS1, the OAS family has been neglected as drug targets, but their role in drug targets still has a lot of room for development. Recent studies have found that Asp75, Tyr230 and Gln229 residues in OAS1 interact with each other (51), which provided value for the development of OAS1 inhibitors.

In addition, the KEGG analysis found that RIG-I-like receptor signaling pathway was enriched. Interestingly, in another study, it was also found that LGALS9 as the prognostic marker of pancreatic cancer was also enriched in RIG-I-like receptor signaling pathway and Cytosolic DNA-sensing pathway (52). A previous study has found that up-regulation of RIG-I-like receptor signaling pathway can promotes migration and invasion of non-small cell lung cancer (53). However, the mechanism of this pathway in pancreatic cancer has not been elucidated. Following viral infection, researchers have discovered that tumor cells mimic innate immunity pathways (54). Activation of viral RNA recognition molecules (RIG-I and MDA5) in tumor cells induced immunogenic cell death (55, 56). Radiation and chemotherapy activate the RIG-I-like receptor pathway, which activates RIG-I, resulting in the physiologic responses to radio-/chemotherapy as an antiviral program (57).

Several limitations in our study should be noted. First, the sample size of pancreatic cancer in TCGA database are significantly smaller than those of other types of cancer, and some clinical data are missing, so it is necessary to expand the sample size for verification. Second, the mechanism of OAS1 in the development of pancreatic cancer was not explored in this study. the current findings identified significant characteristics associated with OAS1 prognosis only by exploring the hypothesized mechanisms. Although the possible mechanism of OAS1 can be preliminarily determined by the analysis of GO, KEGG and GSEA, it still needs further experiments to verify. This is of certain concern, as significant functional and physiological validations would be needed to propel that impressive dataset to the level of relevant biological insight. Finally, our study initially considers that OAS1 may be a potential therapeutic target, but this needs to be confirmed by further experiments.

In conclusion, the high expression of OAS1 is associated with poor prognosis of pancreatic cancer. OAS1 is a valuable prognostic factor in pancreatic cancer. Its close relationship with immune infiltration revealed that OAS1 may also be a potential therapeutic target, but further experimental confirmation is needed.

## Data Availability Statement

The datasets presented in this study can be found in online repositories. The names of the repository/repositories and accession number(s) can be found in the article/**Supplementary Material**.

## Ethics Statement

The studies involving human participants were reviewed and approved by the Ethics Committee of the 900th hospital of the People’s Liberation Army Joint Logistics Support Force. The patients/participants provided their written informed consent to participate in this study.

## Author Contributions

Study concept and design, all. Acquisition of data, LL, HW, JF, and DL. Analysis and interpretation of data, LL, HW, JF, BL, and JZ. Drafting of the manuscript, LL and HW. Contributed to study supervision and critical revision of the manuscript, all. All authors contributed to the article and approved the submitted version.

## Funding

This study was supported by grants from Startup Fund for scientific research, Fujian Medical University (Grant number: 2019QH1285), the 900th hospital of the Joint Logistics Support Force Fund (Grant Number:2020Z12), the 900th hospital of the Joint Logistics Support Force Fund (Grant Number:2020Q02), and Guiding Project of Social Development of Fujian Province (Grant Number: 2021Y0062).

## Conflict of Interest

The authors declare that the research was conducted in the absence of any commercial or financial relationships that could be construed as a potential conflict of interest.

## Publisher’s Note

All claims expressed in this article are solely those of the authors and do not necessarily represent those of their affiliated organizations, or those of the publisher, the editors and the reviewers. Any product that may be evaluated in this article, or claim that may be made by its manufacturer, is not guaranteed or endorsed by the publisher.
