# Supplementary material for: Overexpression of OAS1 Is Correlated With Poor Prognosis in Pancreatic Cancer
Source: Front Oncol. 2022 Jul 11;12:944194. doi: 10.3389/fonc.2022.944194 (PMC9309611; doi:10.3389/fonc.2022.944194)
Supplement: Supplementary file 1 [file Table_1.docx]

Supplementary Material

# Supplementary Table 1. Gene ontology enrichment analysis

| Category | Term | Count | P-value |
| --- | --- | --- | --- |
| BP | GO:0060337~type I interferon signaling pathway | 21 | 5.73E-35 |
| BP | GO:0009615~response to virus | 20 | 1.03E-27 |
| BP | GO:0051607~defense response to virus | 21 | 6.35E-26 |
| BP | GO:0045071~negative regulation of viral genome replication | 11 | 1.19E-16 |
| BP | GO:0060333~interferon-gamma-mediated signaling pathway | 10 | 3.18E-12 |
| BP | GO:0045087~innate immune response | 14 | 2.94E-09 |
| BP | GO:0035456~response to interferon-beta | 5 | 1.83E-08 |
| BP | GO:0032480~negative regulation of type I interferon production | 5 | 3.76E-06 |
| BP | GO:0035455~response to interferon-alpha | 4 | 5.11E-06 |
| BP | GO:0034341~response to interferon-gamma | 4 | 8.32E-05 |
| BP | GO:0034340~response to type I interferon | 3 | 2.61E-04 |
| BP | GO:0035457~cellular response to interferon-alpha | 3 | 4.45E-04 |
| BP | GO:0032481~positive regulation of type I interferon production | 4 | 7.99E-04 |
| BP | GO:0032727~positive regulation of interferon-alpha production | 3 | 8.10E-04 |
| CC | GO:0005829~cytosol | 34 | 2.92E-10 |
| CC | GO:0005737~cytoplasm | 37 | 7.11E-07 |
| MF | GO:0003725~double-stranded RNA binding | 9 | 4.58E-11 |
| MF | GO:0001730~2'-5'-oligoadenylate synthetase activity | 4 | 1.79E-07 |
| MF | GO:0004386~helicase activity | 6 | 1.37E-05 |
| MF | GO:0005515~protein binding | 49 | 1.52E-05 |
| MF | GO:0003727~single-stranded RNA binding | 4 | 5.00E-04 |
| MF | GO:0005524~ATP binding | 14 | 2.28E-03 |

BP, biological processes; CC, cellular components; MF, molecular function; GO, Gene ontology

**、**
